# From tradition to progressiveness: Analyzing Thailand’s image on youtube amid post-cannabis legalization

**DOI:** 10.1371/journal.pone.0317506

**Published:** 2025-02-07

**Authors:** Ibtesam Mazahir, Smith Boonchutima, Safeena Yaseen

**Affiliations:** 1 Department of Social Sciences, Mohammad Ali Jinnah University, Karachi, Pakistan; 2 Postdoctoral Research Fellow C2F The Second Century Fund, Faculty of Communication Arts, Chulalongkorn University, Bangkok, Thailand; 3 Center of Excellence in Communication Innovation for the Development of Quality of Life and Sustainability, Faculty of Communication Arts, Chulalongkorn University, Bangkok, Thailand; 4 Department of Media Studies, Bahria Univeristy, Karachi, Pakistan; Middle Tennessee State University Jennings A Jones College of Business, UNITED STATES OF AMERICA

## Abstract

This research examines the depiction of Thailand on YouTube subsequent to the nation’s decision to legalize recreational marijuana in June 2022. By analyzing 57 video clips recorded between March and September 2022, we investigate the evolution of Thailand’s digital image after the legalization of cannabis in the country. The outcomes of our study demonstrate a predominantly favorable account, highlighting the incorporation of marijuana into Thai society, its therapeutic capabilities, and its potential as a lucrative economic commodity. In intimate connection to elements of the social, physical, and cultural components, the emotional dimension developed as a prevailing theme. Thai residents and government officials were the main influencers in shaping the discussion, representing the prevailing public opinion in support of the choice to legalize marijuana. The prevailing media narratives, which included tourism, celebration, and policy, further emphasized the positive perspective on the advantages of legalizing marijuana. This research emphasizes the profound impact of substantial policy changes on a nation’s international reputation, with Thailand depicted as providing a distinctive combination of cultural heritage and contemporary elements.

## Introduction

The global shift in cannabis perceptions has catalyzed policy changes in many nations, including Thailand, a country traditionally recognized for its cultural heritage. In 2018, Thailand became the first Southeast Asian nation to legalize medical cannabis, followed by recreational cannabis legalization in 2022. These legislative changes marked a significant departure from Thailand’s historical prohibitionist stance, shaping its domestic and international image.

Digital platforms like YouTube play a pivotal role in shaping public opinion, offering a global stage for discourse on cannabis legalization. This study explores Thailand’s portrayal on YouTube, examining how the country’s progressive policies are reshaping its global reputation. Specifically, it seeks to understand how this significant policy change influences Thailand’s digital image and contributes to global discussions about cannabis legalization.

The significance of digital media platforms such as YouTube lies in their ability to mold public opinion. By featuring diverse perspectives, these platforms shape narratives that influence international perceptions. For example, platforms like *The Weed Show* and *High Times* inadvertently educate audiences, promoting nuanced discussions about cannabis policies. However, these narratives are often shaped by cultural, policy, and media dynamics that require careful analysis.

This paper examines the multifaceted consequences of marijuana legalization for Thailand, the only Southeast Asian country allowing recreational cannabis use. The study analyzes the interplay between Thailand’s cultural heritage and its emerging progressive policies, using YouTube as a lens to explore the nation’s post-legalization representation. By employing established scientific methodologies [1–3], the research provides a comprehensive and objective analysis of Thailand’s portrayal on YouTube.

Historically, marijuana has played a significant role in Thai culture, serving medicinal and culinary purposes for centuries. Its prohibition in 1935 marked a shift in its perception as a hazardous substance. Renewed interest in cannabis’ medical potential in the 2000s spurred illegal production until Thailand legalized medical cannabis in 2018. The landmark legalization of recreational cannabis in June 2022 removed marijuana from the list of hazardous substances, spurring broader consumption and a surge in marijuana-related tourism [4].

These developments have profoundly influenced Thailand’s portrayal on YouTube, the most widely used video-sharing platform globally. YouTube’s role as a global platform allows nations to showcase their evolving identities and engage with diverse audiences. Analyzing Thailand’s portrayal following cannabis legalization offers valuable insights into international perceptions and strategic positioning in the post-legalization era.

Thailand’s identity has historically been associated with renowned tourism destinations, such as pristine beaches, exquisite cuisine, and vibrant cultural heritage. Cannabis legalization has the potential to expand this image by incorporating novel elements of progressive policies and economic opportunities. This study examines how Thailand’s evolving identity is depicted on YouTube, emphasizing the convergence of tradition and modernity.

YouTube offers a unique medium for capturing public responses and cultural portrayals. Unlike textual media, video content provides a richer understanding of legalization’s social and cultural effects. By leveraging these insights, this research aims to guide future efforts to enhance Thailand’s international reputation and foster intercultural collaboration.

Through this analysis, we aim to comprehensively understand Thailand’s portrayal on YouTube following cannabis legalization. These insights will help refine Thailand’s global narrative, highlighting its cultural depth and progressive strides.

## Material

In the current age of the convergence of globalization and digitalization, the perception of a country has evolved beyond being only a representation of its geographical and cultural characteristics. Through its evolution, it has become a strategic asset, a brand that effectively conveys its beliefs, advancements, and ambitions to a worldwide audience [5]. YouTube and similar worldwide media platforms have become major contributors in shaping and distributing these pictures due to their extensive reach and effect [6,7]. This research investigates the intricate dynamics of nation branding, the influential capacity of media, and the particular course of Thailand’s image following the legalization of marijuana, with a focus on its representation on YouTube.

National image refers to the overall perception that individuals and groups hold about a nation, which is a multifaceted concept influenced by several elements. Scholars have put forward several frameworks to elucidate the process by which these perceptions are developed. Yang and colleagues [8] present a succinct classification of two main processes: direct and indirect experience. Direct experience encompasses firsthand interactions, travel, and actual usage of products, whereas indirect experience encompasses referrals, media coverage, government advertising, and cultural portrayals. The impact of both forms of experience on views is significant, and comprehending these processes is crucial for nations aiming to enhance their worldwide reputation.

Similar to organizational bodies, each nation has a reputation that it must effectively manage and maintain. Another author [5] contends that reputation management, which was formerly linked to organizations, has now encompassed countries, exerting a substantial impact on global capital movements [9]. A favorable national image has the potential to stimulate tourism, trade, investment, and even diplomatic relations [10]. In 1996, Simon Anholt introduced the concept of “Nation Branding,” which emphasizes the need of carefully constructing and controlling a country’s image to effectively connect with both local and international stakeholders [11].

The national image is the outcome of the interplay among four dimensions: 1-) National identity refers to the fundamental principles, convictions, and inherent traits of a nation, 2-) Nation brand identity refers to the deliberate and controlled portrayal of a country’s image. 3-) Nation brand communication refers to the media and messages used to convey a country’s image. 4-) Nation brand experience refers to the interactions that individuals have with a country, including tourism, trade, and education [12].

National image is a complex concept that may be comprehended in four distinct dimensions: functional, normative, aesthetic, and sympathetic. This dimension pertains to a nation’s performance in domains such as economy, technology, and education. This component pertains to a nation’s principles and convictions, including its dedication to democracy, human rights, and environmental preservation. Aesthetic dimension pertains to the cultural, artistic, and natural aspects of a territory. Sympathy dimension pertains to the attitudes of a nation’s citizens towards trust, kindness, and hospitality [13].

Furthermore, other authors propose that nation branding should be a cooperative endeavor encompassing all parties involved, such as government, corporations, citizens, and NGOs [14]. Therefore, it is crucial to establish a common vision for the national brand. Authors like Anholt delineated six fundamental aspects of nation branding: governance, human capital, exports, tourism, culture and heritage, and investment and immigration policy [10]. Hence, it is imperative to effectively control these aspects in a synchronized fashion in order to establish a prosperous national brand. Nation branding involves the art of narrating captivating narratives about the core identity, cultural legacy, and principles of a nation [14]. Furthermore, this underscores the need of employing narrative techniques to establish an emotional connection with audiences. Conversely, YouTube has arisen as a novel medium for narrative, exhibiting the inventive acumen and skill of storytellers. Through the skillful integration of oral tradition and the dynamic nature of social media, storytellers effectively engage audiences and convey knowledge [15]. Given Thailand’s status as one of the most sought-after tourist destinations globally, it is intriguing to observe the manner in which international vloggers on YouTube, the largest user-generated video sharing site, depict the country. YouTube has become a potent platform for influencing worldwide opinions, and its vast collection of vlogs provides a distinctive insight into public perceptions of Thailand among tourists and enthusiasts worldwide.

Numerous studies have underscored the significant impact of mass media, particularly in molding opinions of other countries, by emphasizing a distinct link between perceptions and news media coverage [16]. The advent of social media platforms like Facebook, YouTube, Instagram, and Twitter has completely transformed the process of worldwide information sharing. These platforms function as comprehensive real-time multimedia data sources that visitors utilize to record and distribute their experiences. YouTube, a prominent video-sharing network, offers a vast number of user-generated videos on a daily basis, providing a diverse range of content that extends beyond entertainment, encompassing education and the distribution of news. The inclusion of participatory mechanisms, such as comments, renders it an indispensable instrument for both industry and study. The extensive popularity of YouTube has established it as an essential platform for obtaining a wide range of content and expertise [17].

Aside from its video-focused emphasis, YouTube also integrates social networking features that promote user engagement. Registered users have the ability to engage with videos by sharing, rating, and commenting on video content. These interactive elements promote a feeling of community by facilitating comments, thereby stimulating participation and discourse, which in turn can enhance the evaluation of the legitimacy of the material [6]. YouTube is a highly popular online platform, boasting a vast global user base numbering in the billions. YouTube proved to be the most favored social media platform in the United States, with Instagram and Snapchat following closely behind [18]. YouTube remains the preeminent social media platform among teenagers throughout Europe, however its popularity differs among nations, indicating distinct adoption trends [19]. Notably, a substantial number of Thailand’s foreign visitors originate from the United States and Europe [20].

Thailand’s reputation as a worldwide visitor tourism destination has been meticulously crafted over time through the strategic use of advertising campaigns, travel documentaries, and cultural portrayals. Few authors have emphasized the critical role of strategically promoting Thailand’s exotic appeal in attracting international tourists, therefore making a substantial contribution to the country’s foreign exchange reserves [21]. Others argue that Thailand’s reputation as the “Land of Smiles” has played a crucial role in fostering its amiable and inviting image, which has been well received by international audiences [21].

Thailand achieved a significant milestone in Asia in June 2022 by decriminalizing marijuana, therefore allowing for the unrestricted growth and use of this substance. Particularly, the alterations in the social and cultural terrain of the nation. Although marijuana was officially legalized in 2022, its historical background reveals that the marijuana issue in Thailand has been a subject of debate much before Prime Minister Prayut Chan-o-cha assumed office. Cannabis has been utilised in Thailand for millennia, serving both medicinal and culinary uses. This plant was highly esteemed and regarded as an integral component of the local culture at that period. Thailand initiated the implementation of a prohibition law against marijuana in 1935, adopting a law that was subsequently adopted by several countries worldwide. Cannabis was deemed a hazardous substance and subject to rigorous control through regulation. In the 2000s, despite the ongoing prohibition of marijuana, numerous research started to demonstrate its therapeutic capabilities. Illicit marijuana growing operations surged, particularly in rural regions.

Continuing this trend, Thailand became the pioneering nation in Southeast Asia to legalize Cannabis for medicinal purposes. Officially sanctioned by the government, this legislation permitted the study and application of marijuana in the field of medicine. It resurfaced as a subject of discussion in June 2022, when the Thai government eliminated marijuana from the list of hazardous substances, therefore enabling its extensive consumption. A significant change in the nation’s drug laws occurred, leading to a heightened fascination with tourism centered around marijuana [4]. Although most discussion is focused on the possible economic and health advantages of this decision, there have been worries expressed about its impact on Thailand’s well-established reputation. The legalization of this substance has garnered considerable national and international interest, with discourse mostly centered on its possible economic and health consequences [22–25].

Although some studies conducted in the US, Canada, and Portugal have investigated the effects of marijuana legalization, these researches have mostly concentrated on factors unrelated to the possible alteration of a country’s reputation after legalization. Although these studies examine the portrayal of arguments for legalization by the mainstream media and highlight the actors and sources that get substantial media coverage, they do not directly discuss the wider effects on a country’s overall image that arise from the legalization process [26–28].

Likewise, prior research on the representation of Thailand as a tourist destination has frequently focused on country-specific aspects. Another study examined how Thailand is depicted in the English media, with a particular focus on its reputation as a destination for sex tourism [29]. A study conducted by Deng and fellow researchers examined the narratives in Chinese media after the legalization of marijuana, focusing on Thailand’s changing perception as a favored tourism spot [30]. Preceding its legalization, other research has emphasized the endeavors of Thai government authorities to advocate for the therapeutic advantages of marijuana [4]. Despite these observations, there is a dearth of thorough study that comprehensively analyzes the representation of Thailand in the worldwide media environment, particularly since the legalization of marijuana.

Global media platforms reflect and shape global perceptions. Interactive digital media in real-time revolutionizes communication by providing global access and tailored consumption. Analytically optimized user-generated material stimulates active participation. In contrast to conventional one-way communication, which lacks the interactive characteristics of digital media, the dynamic evolution of media stands out. Featuring a staggering 2 billion monthly subscribers, YouTube is a prominent but insufficiently studied social media network [31]. The platform presents a contemporary alternative to conventional media by combining internet and television elements, therefore revealing the inherent inconsistencies between broadcast and limited digital transmission. Beyond user-generated content, YouTube functions as a content hub with robust links to web pages and mainstream media in a linked media ecosystem [32,33].

The media irrefutably plays a significant influence in influencing the perspective and image of a nation. The impression of a country or culture in the eyes of other nations is typically shaped by the mass media coverage of international events [9]. Several stakeholders such as the president, foreign policy advisers, elites, and the media participate in a struggle to influence the narratives that are communicated to the public through the media. These frames have a remarkable impact on the development of public opinion [3].

YouTube has significantly broadened the scope and influence of digital media in the modern era. YouTube, with its worldwide viewership, has the capacity to dramatically shape the perception of a country [7,34]. With its widespread use and integration with other social networking sites, as previously mentioned in this section, it is crucial to comprehend how YouTube presents and presents a country, particularly after major policy shifts like the legalization of marijuana in Thailand.

Varying forms of media content provide distinct viewpoints. In 1972, McCombs and Shaw observed that news media had the ability to establish the public agenda, so shaping the significance that viewers attribute to certain subjects. In contrast, vlogs provide a more intimate viewpoint, frequently offering firsthand observations and personal encounters [35]. YouTube has transformed the process by which passengers select their locations, setting it as an essential instrument for the advancement of the tourism sector. The extensive collection of travel films on this platform offers immersive and captivating experiences, enabling prospective tourists to virtually explore specific locations, fully engage with local cultures, and acquire vital insights into the trip experience. This visual narrative technique has revolutionized the process of organizing trips, enabling tourists to make well-informed choices by relying on genuine and pertinent material provided by vloggers and mainstream media on YouTube [36].

This work integrates the multidimensional Fombrun Country Reputation Index (CRI) that was first established by Passow et al. in 2005. Emotional, Physical, Financial, Leadership, Cultural, and social attraction are the six specific dimensions of attraction included in this assessment. Given its significance to this research, a supplementary aspect, Political Attractiveness, has been incorporated as a subtopic. The incorporation of recommendations provided by [8] serves to further strengthen the comprehensive research strategy. The objective of this study is to address the existing research void by conducting a methodical analysis of the representation of Thailand on YouTube, specifically related to the legalization of marijuana. The focus is on extracting meaning, context, and intent encoded in messages, following Prasad’s method suggestion for a scientific approach and Neuendorf’s explanation of fundamental processes in media content analysis [1,2]. Analysis of content is a useful approach for obtaining meaning from media material. In this method, several factors are analysed, including the subject matter, frequency of reference, contextual “messages” identified by keywords, degree of media impact, and recurrence of specific themes [3,37,38].

Considering these concerns, this article will assess Thailand’s video representation on YouTube both prior to and following the legalization of marijuana in the country. This study seeks to derive significant insights for the future development of Thailand’s national branding strategy by thoroughly analyzing its present image in the eyes of the international community. This work will explicitly focus on the following research inquiries:

- To what extent will the legalization of marijuana affect the portrayal of Thailand on YouTube?
- When portraying Thailand on YouTube in the post-legalization era, which narrative frames do global media outlets predominantly employ?
- How did the narrative surrounding Thailand on YouTube change, both before and after the legalization of marijuana?

For a thorough evaluation of Thailand’s media representation, it is crucial to acknowledge two fundamental basic principles. (a) National Image: This notion includes several aspects that impact the reputation of a country—(b) Media Framing: The notion of framing examines the portrayal of topics in the mass media. This study will be grounded on these principles as its theoretical framework.

## Methods

The primary analysis of this study is on YouTube video material pertaining to marijuana in Thailand. The content encompasses a broad spectrum of sources, with a focus on videos conducted within the specified research time to document the changing views after legalization. With its unparalleled reach and diverse range of content categories, YouTube was selected as the main platform for this research, providing a comprehensive perspective on worldwide opinions of Thailand after the legalization of marijuana. For the dissemination of information and the formation of public opinion, YouTube is among the most significant media platforms. With a staggering two billion monthly logged-in users, YouTube’s extensive reach is unparalleled, establishing it as a fundamental information source for many regarding worldwide events and policy changes. YouTube, unlike other conventional media platforms, has emerged as an indispensable tool for prospective travelers, offering them a plethora of information and comprehensive experiences to facilitate well-informed choices in destinations selection [36]. Therefore, for a nation such as Thailand that is highly dependent on tourism income, it is crucial to examine its image on YouTube. For a subject such as the legalization of marijuana, YouTube provides a diverse range of news stories, personal anecdotes, expert viewpoints, and instructional material, therefore offering a full perspective on public attitude and discussion. Due of its extensive usage and vast range of content, YouTube was selected as the main medium to examine comprehensive depictions of Thailand’s post-marijuana legalization environment.

The study conducted between March 9, 2022, and September 9, 2022, focused on the timeframe preceding and following the formal legalization on June 9, 2022. This timeframe offers an equitable period for analysis. YouTube searches were conducted periodically throughout the six-month study period, ensuring the inclusion of videos that reflect the dynamic and ongoing discussions related to cannabis legalization in Thailand during this time frame. The six-month period offers a well-balanced timeframe for examining Thailand’s representation on YouTube, encompassing both prior to and following the legalization event. The selected time period enables a thorough evaluation of the impact of marijuana legalization on Thailand’s YouTube image, including both the early responses and the enduring consequences of this momentous occurrence. Within the framework of a policy shift like the legalizing of marijuana, this specific time period enables a more thorough examination of the public’s reaction to the event immediately following its execution. Moreover, the six-month data covers the initial stage when the general public and media are starting to investigate the consequences of the new policy. During this period, there is a significant level of public interest, and the material generated, whether from news articles, vlogs, or personal anecdotes, might mirror evolving opinions and depictions that might impact the choices made by travelers.

In this study, the significance of keyword selection resides in its capacity to encompass a broad spectrum of material, ranging from official news articles to personal Vlogs, guaranteeing a thorough analysis. Keywords were determined through a combination of literature review, preliminary search testing and feedback from 50 random international tourists. These participants were consulted to identify search terms they might use when seeking information about cannabis legalization in Thailand. This process ensured that the selected keywords—‘Thailand,’ ‘marijuana,’ ‘legal marijuana,’ ‘marijuana tourism,’ ‘cannabis,’ ‘drugs,’ and ‘legalization of marijuana’—captured a diverse range of relevant YouTube content. This methodical strategy was thereafter employed to gather pertinent video footage. To guarantee precise video gathering, each keyword was inputted into YouTube’s Advanced Search Options. Therefore, throughout the first stage, a total of 57 videos were discerned as pertinent.

Out of the 57 clips examined, several videos were found to be interrelated, particularly those from news media and vloggers that addressed the same topic or included references to the same incident. Recognition of these snippets is crucial for comprehending the constructed narratives and the reciprocal influence of the information. Specifically, international vloggers have a crucial role in influencing Thailand’s perception after the legalization of marijuana by offering foreign viewpoints. They can be classified as pro-legalization vloggers who enthusiastically promote marijuana usage and share pleasant experiences, neutral vloggers who present factual information without bias, and critics or anti-legalization vloggers who express concerns about the consequences of legalization. Their presence facilitates a wider and more worldwide examination, offering thorough understanding of how overseas audiences perceive the legalization of marijuana in Thailand.

The authors declare that the principles of ethical and professional conduct have been followed. Although the research does not involve human participants the study was approved by Office of the Research Ethics Review Committee for Research Involving Human Subjects: The Second Allied Academic Group in Social Sciences, Humanities and Fine and Applied Arts under the COA No. 339/66.

### Criteria for video inclusion

1. ***Relevance****:* Videos that directly discuss Thailand’s cannabis legalization or its implications were included. This could range from news reports, expert panels, Vlogs, or educational content.
2. ***Language****:* Given the study’s focus on international perceptions, only videos in English or those with English subtitles were considered to ensure the researchers’ understanding and accurate analysis.
3. ***Duration****:* Videos ranging from 2 to 30 minutes were prioritized. This range was chosen to exclude very short clips that might lack depth and very long videos that might be less accessible to the general audience.
4. ***Date of Upload****:* Videos uploaded between March 9, 2022, and September 9, 2022, were considered to capture the evolving perceptions post-legalization.

### Criteria for video exclusion

1. ***Off-topic Content****:* Any video that merely mentioned Thailand or cannabis in passing but did not delve into the topic of legalization was excluded.
2. ***Non-English Content****:* Videos without English subtitles or translations were excluded to ensure accurate content analysis.
3. ***Duplicate Content****:* If multiple channels or users uploaded the same content, only one instance was considered to avoid redundancy.

By adhering to these criteria, the study aimed to ensure a focused yet comprehensive analysis of YouTube content related to Thailand’s cannabis legalization.

A codebook, essential for ensuring consistent categorization, was developed based on methodologies from previous studies, allowing for systematic video content analysis [39,40]. It is divided into:

1. ***Formal Categories****:* Providing general information about the clips.
2. ***Content Categories****:* Derived from literature and research questions to aid analysis.

The details, including variables and categories, have been included in the codebook. This systematic approach objectively analyzes Thailand’s portrayal, especially post-cannabis legalization.

To ensure consistent interpretation across different coders, the study employed Holsti’s method, a reliable measure that reflects the agreement ratio in coding decisions [41]. This method reflects the number of agreements per total number of coding decisions. The coefficient of reliability (C.R.) provides a formula for calculating percent agreement. The formula for determining the reliability of data in terms of percentage of agreement is $\left(\text{2M}\right)\text{/}\left(\text{N1}+\text{N2}\right)\left(\text{2M}\right)\text{/}\left(\text{N1}+\text{N2}\right)$where M is the number of coding decisions on which two coders agree, and N1 and N2 refer respectively to the total number of coding decisions by the first and second coder. After conducting intercoder reliability for the 42 variables, a Holsti coefficient of 0.93 was achieved, indicating high intercoder reliability, with each category obtaining a reliability coefficient of no less than 0.89.

Before initiating the coding procedure, a pre-test is undertaken to identify frames when employing framing analysis on the sample. For this pre-test, the researcher randomly selected 20% of the total clips and meticulously documented all discernible frames within the video clips. Subsequently, the researcher categorized these identified frames by grouping terms with analogous meanings into distinct categories. These categorized frames are then integrated into the comprehensive codebook to serve as a systematic reference during the subsequent coding process.

## Result

The legalization of cannabis in Thailand has prompted mainstream media and digital platforms, like YouTube, to play a pivotal role in conversations, debates, and depictions of this significant policy shift. This part delves into the findings of our methodical investigation, elucidating the manner in which Thailand’s perception has been built within this novel setting.

The examined videos basically belong to two genres: News and Vlogs. Out of the entire clips examined, 52.6% were news segments sourced from several prominent media organizations worldwide, totaling 30 clips. Vlogs, including personal video blogs produced by people and travel vloggers, accounted for 47.4% of the clips, totaling 27. Although mainstream media has extensively covered the subject, individual content creators and the general public have also shown considerable interest in discussing and sharing their viewpoints on cannabis legalization, as seen by the similar percentages of these two categories.

A monthly distribution of YouTube clips pertaining to Thailand’s cannabis legalization is shown in Table 1, demonstrating the changing pattern in content production over time. In both March and April, there was a comparatively low level of interest, with just three clips apiece, or 5.3% of the total for those months. An incremental rise was observed in May, with the inclusion of five clips, accounting for 8.8% of the full. Yet, June saw a substantial increase, with 21 clips representing 36.8%. This spike can be ascribed to the official date of legalization in June, which probably triggered intensified debates and media attention. Following the legalization, there were eight recorded clips in July, accounting for 14% of the total, and 15 in August, or 26.3%, indicating prolonged interest. However, by September, the coverage had reduced to a mere two clips, accounting for 3.5% of the overall amount.

**Table 1:** Monthly Breakdown of YouTube Clips.

Month	Count	Percentage
March	3	5.3%
April	3	5.3%
May	5	8.8%
June	21	36.8%
July	8	14.0%
August	15	26.3%
September	2	3.5%

According to the statistics shown in Table 1, the most significant levels of interest and conversation around the legalization of cannabis took place during and immediately after the month of legalization. This observation is corroborated by the fact that most films were uploaded during the period preceding the country’s declaration of legalization in June.

Examining the interconnectedness of these clips, other videos from mainstream media and vloggers tackled comparable subjects or made reference to the same events. In order to comprehend the manufactured narratives and the reciprocal impact of the information supplied, it is essential to acknowledge these relationships. The contribution of international vloggers in influencing public opinions of Thailand after its legalization is significant, as they provide a wide range of perspectives. Their content can be classified into three categories: pro-legalization vloggers who fervently advocate for cannabis use and share favorable experiences; neutral vloggers who present objective facts without prejudice; and critics or anti-legalization vloggers who voice apprehensions about the consequences of legalization. This variant enables a more comprehensive and worldwide analysis of how overseas audiences view the legalization of cannabis in Thailand.

The data depicts the distribution of YouTube videos before and after the legalization of cannabis in Thailand, demonstrating a distinct increase in content creation by YouTubers subsequent to this notable public policy shift. Significantly, a considerable percentage of the videos, namely 81%, were posted on YouTube subsequent to the announcement of legalization, but only a marginal fraction of media discussions (19%) discussed the subject matter in advance. These results emphasize the small amount of media attention given to the legalization of cannabis before the announcement, indicating that the decision served as a stimulus for heightened public discussion.

The categorization of Thailand’s image on YouTube across several aspects is presented in Table 2, illustrating the several approaches in which the subject has been addressed. The emotional aspect was the most prominent, as evidenced by 51 video (89.5%) evoking emotions or thoughts linked to Thailand’s decision to legalize cannabis. The heightened emotional involvement seen suggests a robust correlation between the legalization policy and the prevailing public opinion, therefore indicating a positive and enthusiastic attitude towards its prospective advantages.

**Table 2:** Dimensions of Thailand’s Image.

News Dimension	Number of Count	Percentage of Coverage on YouTube
Emotional	51	89.5%
Physical	44	77.2%
Leadership	32	56.1%
Financial	32	56.1%
Cultural	40	70.2%
Social	47	82.5%
Political	29	50.9%

Furthermore, with the emotional content, the social and physical components were widely showcased, as seen by 47 videos (82.5%) that addressed the concrete features of Thailand’s landscape and its practical consequences for society. Frequently, these debates centered on the anticipated consequences of legislation on tourism, public health, and community welfare. Cultural elements were particularly noteworthy, being present in 40 clips (70.2%), emphasizing the incorporation of cannabis into Thailand’s abundant cultural legacy and customary rituals.

A total of 32 clips (56.1%) specifically addressed the leadership, financial, and political aspects, which were discussed in relation to government regulations and economic prospects associated with the cannabis sector. Moreover, the political aspect, depicted in 29 short films (50.9%), highlighted the changing regulatory environment and the Thai government’s influence on public opinion and legislation concerning cannabis.

The presented distribution highlights the complex and diverse character of the discussion on the legalization of cannabis in Thailand, encompassing both the concrete and abstract consequences of the legislation. Furthermore, it emphasizes that the story goes beyond the substance itself and includes wider concepts of social transformation, cultural identity, and economic prospects. The combined knowledge acquired from these videos offers a thorough comprehension of how Thailand’s perception is being reconfigured following this significant ruling, encompassing both local and international viewpoints on the legalization of cannabis.

A comprehensive examination of the tone linked to each aspect of Thailand’s representation in the context of cannabis legalization is shown in Table 3. A remarkable 73.7% of the clips displayed a positive tone in the emotional component, reflecting a robust public attitude that corresponds to hope and endorsement of the legalization effort. In contrast, a mere 7% of the short films expressed a negative sentiment, underscoring the largely positive reaction of the policy change among both content producers and viewers.

**Table 3:** Tone of the Dimensions.

Tone of Dimension	Emotional	Physical	leadership	Financial	Cultural	Social	Political
Positive	73.7%	68.4%	42.1%	43.9%	59.6%	66.7%	29.8%
Negative	7.0%	5.3%	3.5%	5.3%	1.8%	5.3%	5.3%
Ambivalent	7.0%	–	7.0%	5.3%	3.5%	8.8%	10.5%
Neutral	1.8%	3.5%	3.5%	1.8%	5.3%	1.8%	5.3%
Not Found	10.5%	22.8%	43.9%	43.9%	29.8%	17.5%	49.1%

The physical dimension revealed a favorable inclination, as 68.4% of the videos portrayed a hopeful viewpoint on the concrete elements of Thailand, including its scenery, infrastructure, and prospects associated with cannabis tourism. This optimistic portrayal implies a conviction in the capacity for progress and advancement inside the nation subsequent to the legalization.

The topics of leadership and finance received comparable favorable attention, with 42.1% and 43.9% of the clips respectively highlighting the beneficial impact of government policies and the economic opportunities linked to the cannabis sector. These findings highlight the apparent congruence between leadership efforts and public opinion, promoting a story of advancement and possible advantages for the country.

From a cultural perspective, the videos also exhibited a preference for a favorable depiction, as 59.6% of them revealed an optimistic perspective on the incorporation of cannabis into Thailand’s diverse cultural fabric. In close succession, the social aspect emerged, as 66.7% of the videos expressed views that emphasize the social consequences of legalization, including the enhancement of community welfare and the advantages for public health.

Notably, the political aspect received the least favorable coverage, as only 29.8% of the clips depicted it in a good light. It is possible that this reflects persistent worries or uncertainty over the regulatory framework pertaining to cannabis. The overall negative tone across all dimensions remained rather restrained, with the leadership component exhibiting the greatest level at a mere 3.5%.

The levels of ambivalence, neutrality, and lack of a distinct tone differed among dimensions. Notably, the social and political dimensions exhibited significant ambivalence, with rates of 8.8% and 10.5% respectively. These findings suggest that although there is a general sense of hope, there are also notable subtleties and intricacies in public sentiment, namely about the political environment and its consequences for the future of cannabis legislation in Thailand.

These results demonstrate that although the general attitude is mostly positive in most aspects, there are still important aspects, especially in political discussions, that require further investigation and comprehension. This extensive analysis not only emphasizes the positive response of cannabis legalization but also identifies the areas where concerns and uncertainty persist, indicating a complex public discussion that is still developing.

An exhaustive summary of the general tone linked to the video clips examining Thailand’s cannabis legalization is provided in Table 4. A significant majority, comprising 43 clips (75.4%), depicted the decision in a positive manner, suggesting a robust and wide-ranging positive reception among YouTube content creators and users. The strong positive sentiment indicates that a significant number of people saw the legalization as a forward-thinking measure for Thailand, emphasizing its possible advantages for tourism, health, and economic growth.

**Table 4:** Overall tone of the video clips.

Overall Tone of the Video	No. of Count	Percentage
Positive	43	75.4%
Negative	4	7.0%
Ambivalent	9	15.8%
Neutral	1	1.8%

By contrast, a mere 4 clips (7%) expressed a negative tone, indicating a negligible amount of criticism or adverse opinions about the policy overhaul. The minimal presence of negative sentiment indicates a widespread agreement among content creators that the legalization of cannabis is a desirable decision for the nation.

Furthermore, 9 clips (15.8%) had ambiguous tones, which are characterised by a lack of significant inclination towards either positive or negative sentiments. This implies that a segment of the material presented an impartial or ambivalent viewpoint, recognizing both the possible advantages and the difficulties that may result from the legalization. Ambivalence of this nature might be advantageous, since it indicates a more sophisticated comprehension of the intricacies associated with the matter.

Only one clip (1.8%) exhibited neutral tones, indicating a lack of significant sentiment. This limited availability further underscores the prevailing inclination towards optimism in the discussion that surrounds Thailand’s cannabis policy.

The data presented in Table 4 strongly supports the notion that the general public opinion toward the legalization of cannabis in Thailand is very positive. The prevailing upbeat tone in most of the videos not only emphasizes the optimistic nature of this policy reform but also emphasizes Thailand’s potential to become a frontrunner in the cannabis sector in the region. The positive depiction is likely to have consequences for tourism, economic development, and global image, ultimately adding to a larger story of advancement and creativity in Thai society.

**Fig 1:**
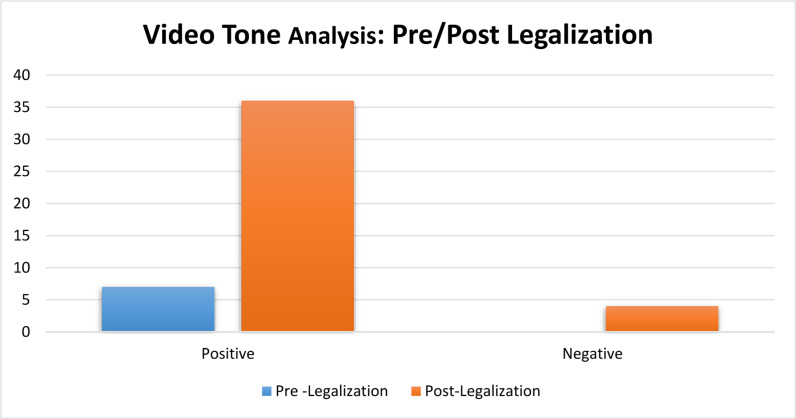
Analysis of Video Tones: Pre- and Post-Legalization.

Fig 1 depicts a notable difference in the tone of videos that address the legalization of cannabis in Thailand, emphasizing the profound impact of this policy adjustment. Upon the legalization of cannabis, there was a significant surge in videos that exhibited a positive tone, suggesting a more favorable public opinion of the legalization. Before legalization, there were no recordings of negative-toned videos. However, after legalization, a small number of such videos surfaced, expressing critical viewpoints and concerns about the consequences of the policy. This research highlights that the legalization of video content has had a significant impact on the attitudes conveyed, leading to a noticeable rise in favorable depictions and a least occurrence of unfavorable criticism. The observed fluctuations in sentiment indicate a wider evolution in public and media involvement with the subject, promoting a more vibrant discourse after the implementation of the policy.

Comprehending the primary voices that influence this storyline is essential for understanding the developing intellectual discussion. The talks revealed that Thai civilians were the most often cited participants, closely followed by government officials, suggesting a narrative with a strong foundation in local viewpoints. Table 5 provides a comprehensive overview of the players who predominantly depicted the legalization of cannabis in a favorable manner, highlighting its prospective advantages and societal consequences.

**Table 5:** Most Quoted Actors in YouTube Clips.

First Quoted Actor	%	Second Quoted Actor	%	Third Quoted Actor	%
Thai Citizens	42.1	Not Mentioned	28.1	Not Mentioned	64.9
Thai Government Officials	26.3	Thai Cannabis, Growers, Handlers, Sellers & Advocate	19.3	Thai Cannabis, Growers, Handlers, Sellers & Advocate	8.8
International Citizens	10.5	International Media Source	15.8	International Citizens	7
Thai Cannabis, Growers, Handlers, Sellers and Advocate	10.5	Thai Citizens	14.0	International Media Source	5.3
International Media Source	5.3	International Tourists/Citizens	8.8	Thai Government Officials	3.5
Thai Media Source	3.5	Thai Government Officials	5.3	Thai Academicians and Researchers	3.5
Thai Media Source	3.5	Thai NGO	1.8	Thai Citizens	3.5
Thai Law Enforcement Agencies	1.8	Thai Academicians and Researchers	1.8	Thai Media Source	1.8
		Thai Media Source	1.8	International Government Officials	1.8
		International Government Officials	1.8		
		International Artists	1.8		

Table 5 offers a detailed look at the actors most frequently quoted in the YouTube clips related to Thailand’s cannabis legalization. The table is structured to show the top three quoted actors in the clips.

- *First Quoted Actor.* Thai citizens dominate the discourse as the primary quoted actors in 42.1% of the clips. This suggests that the voices of the local Thai populace are central to the narrative. Following them, Thai government officials are quoted as the primary source in 26.3% of the clips, indicating the government’s active role in shaping the discourse. Other notable actors include international citizens, Thai cannabis growers, handlers, sellers, advocates, and international media sources, with percentages ranging from 10.5% to 5.3%.
- *Second Quoted Actor.* Interestingly, in 28.1% of the clips, a second actor is not mentioned, suggesting a singular narrative focus in these videos. However, where a second actor is present, Thai cannabis growers, handlers, sellers, and advocates are quoted in 19.3% of the clips, followed by international media sources (15.8%) and Thai citizens (14.0%).
- *Third Quoted Actor.* A significant 64.9% of the clips do not mention a third actor, indicating a more streamlined narrative in these videos. Among the clips that mention a third actor, Thai cannabis growers, handlers, sellers, and advocates are quoted in 8.8% of the clips, followed closely by international citizens (7.0%) and international media sources (5.3%).

The analysis of Table 5 indicates that the discussion on YouTube on Thailand’s cannabis legalization is predominantly influenced by Thai citizens and government officials. These two groups play a crucial role in influencing public opinion, both voicing robust endorsement for legalization as an innovative and forward-thinking action for the nation. Their participation underscores a regional agreement on the possible advantages of cannabis legalization, including as economic prospects, improved public health, and cultural acknowledgment of age-old customs.

As the story progresses, it becomes evident that various perspectives are increasingly assuming a substantive part in the discourse. An expanding number of stakeholders from the cannabis sector, including producers, handlers, and advocates, are expressing their viewpoints, highlighting the economic opportunities and social consequences of cannabis as a legally traded product. By offering a deep grasp of the operational and regulatory issues inside the rapidly growing cannabis industry, their perspectives enhance the discussion.

Moreover, the incorporation of global media and individuals expands the range of the debate even further. Frequently, international media outlets provide comparison evaluations from other nations that have had comparable legalization procedures, therefore providing significant lessons and perspectives. Such a global outlook not only places Thailand’s specific circumstances in context but also promotes a more thorough analysis of the consequences of legalization on both domestic and global levels.

**Fig 2:**
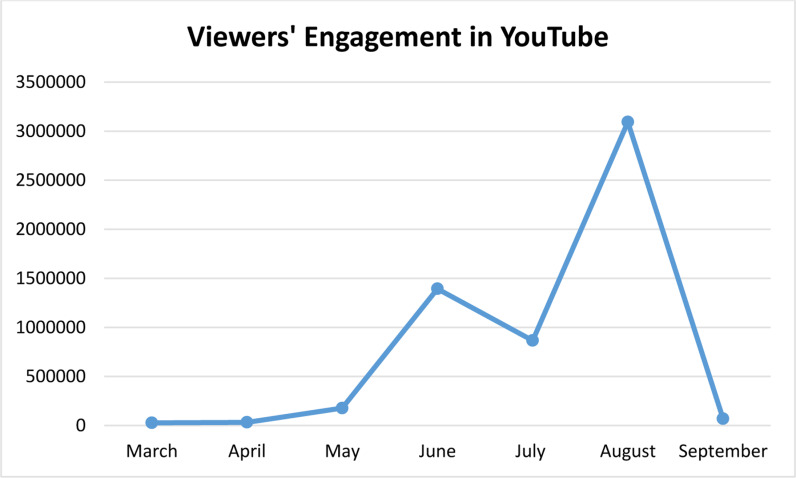
Audience Engagement in YouTube over the months.

Fig 2 depicts the patterns of viewer interaction with material pertaining to Thailand’s cannabis legalization over a span of many months, emphasizing the substantial influence of this regulation shift on the attention of online audiences. The months preceding and succeeding the legalization establish crucial benchmarks for examining changes in viewer involvement.

Throughout the pre-legalization period, which spanned from March to May, the total number of views remained somewhat low, indicating a restricted level of public interest. Nevertheless, a conspicuous increase in May signifies a rising interest and expectation associated with the imminent legalization, implying that the announcement was creating excitement even prior to the designated date.

By stark contrast, the period after legalization, namely from June to September, saw a very significant increase in overall viewership. June represented a crucial turning point, as evidenced by an impressive 1,393,787 views documented, highlighting a substantial surge in public participation and interest. This surge may be ascribed to the official declaration of legalization, which probably ignited extensive debates and motivated numerous users to search for material pertaining to the subject.

The pattern persisted in the following months, as the period from July to September consistently garnered a higher level of viewership in comparison to the period before legalization. Significantly, August stood out as a remarkable month, attaining an amazing 3,093,089 views. This enduring curiosity indicates that the legalization of cannabis not only attracted first notice but also continued to generate continuing debates and investigations of its consequences in Thailand.

In Table 6, the YouTube videos are classified according to the media frames used to analyze Thailand’s cannabis legalization. Through the analysis of these frames, we get an understanding of how different elements of legalization, including economic advantages, cultural importance, health consequences, and regulatory obstacles, are shown in particular types of content. Adopting this classification will facilitate comprehension of the dominant narratives that influence public opinion and involvement with the legalization of cannabis in Thailand. Collectively, the data emphasizes a distinct link between changes in regulations and increased viewer involvement, demonstrating how substantial policy changes can stimulate public discussion in the digital realm.

**Table 6:** Media Frames.

Frames	Count	Percentage	Frames	Count	Percentage
Drug Trafficking	7	12.3%	Social and Cultural	20	35.1%
Stereotyping	2	3.5%	Legal	36	63.2%
Tourism	38	66.7%	Travel Advisory	14	24.6%
Celebration	37	64.9%	Competition	15	26.3%
Health and Wellness	31	54.4%	Human Interest	33	57.9%
			Policy	24	42.1%

Table 6 categorizes the YouTube clips based on the media frames employed to discuss Thailand’s cannabis legalization.

- *Drug Trafficking.* This frame appears in seven (12.3%) clips, suggesting that a segment of the content links cannabis legalization to broader issues of drug trafficking in Thailand.
- *Stereotyping.* Only two clips (3.5%) employ a stereotyping frame, indicating minimal content that might pigeonhole or generalize the Thai populace or cannabis users.
- *Tourism.* A dominant frame, appearing in 38 (66.7%) clips, underscores the potential of cannabis tourism in Thailand post-legalization.
- *Celebration.* This frame, present in 37 (64.9%) clips, captures the collective euphoria or positive sentiment surrounding the legalization.
- *Health and Wellness.* Over half (31) of the clips (54.4%) employ this frame, emphasizing the medicinal and wellness benefits of cannabis.
- *Social and Cultural Frame.* This frame, present in 20 (35.1%) clips, delves into the societal and cultural implications of cannabis legalization in Thailand.
- *Legal Frame.* A significant 36 (63.2%) clips employ this frame, highlighting the decision’s legal nuances, regulations, and implications.
- *Travel Advisory.* Found in 14 (24.6%) clips, this frame offers guidance or cautions to potential tourists or travelers regarding cannabis use in Thailand.
- *Competition.* This frame, present in 15 (26.3%) clips, possibly discusses Thailand’s position in the global cannabis market or its competitive edge post-legalization.
- *Human Interest.* Over half of the clips (57.9%) employ this frame, focusing on personal stories, experiences, or the human aspect of legalization.
- *Policy.* Found in 24 (42.1%) clips, this frame delves into the policy decisions, strategies, and government stance on cannabis legalization.

Table 6 demonstrates that the discussion on YouTube over Thailand’s regulation of cannabis is diverse and intricate. Despite the dominance of frames relating to tourism, celebration, and the legal features of cannabis in the narrative, there is also significant emphasis on health benefits, human interest tales, and policy debates, according to the analysis.

The significance of tourism and celebration frames exemplifies the enthusiasm around Thailand’s capacity as a cannabis tourism center, emphasizing the cultural assimilation of cannabis into its local customs and culinary traditions. Furthermore, the legal framework emphasizes the importance of regulatory modifications and their consequences for both inhabitants and tourists.

Moreover, the incorporation of health benefits frames demonstrates the favorable views of cannabis as a therapeutic asset, in line with the increasing worldwide narratives around medicinal cannabis. Personal interest stories offer a unique perspective by highlighting human experiences and the effects on communities, therefore enhancing the conversation.

Nevertheless, the inclusion of frames such as drug trafficking and travel advisories suggests that the material also carefully acknowledges and tackles important obstacles and apprehensions associated with the decision to legalize. The purpose of these frames is to remind us of the intricate nature of cannabis policy, which includes the requirement of effective communication regarding legal rules to avoid any misinterpretations, particularly among visitors.

**Fig 3:**
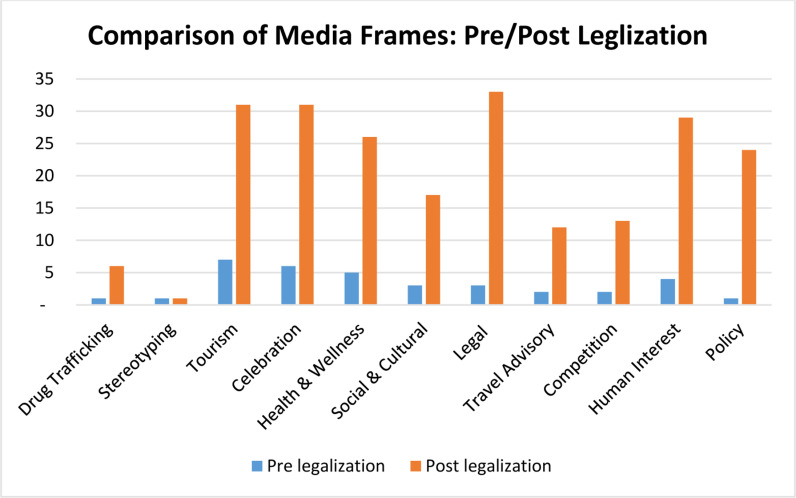
Media Frames: Pre/Post Legalization.

Fig 3 classifies the comparative analysis of media frames used to depict Thailand’s cannabis legalization, emphasizing the distribution of frames before and during the regulatory reform. Each frame depicts a distinct viewpoint or emphasis, revealing the developing nature of debates on the subject in reaction to legalization.

After legalization, the data indicates a significant shift in conversation, with specific frames, such as wellness and health advantages, becoming more prominent. Prior to legalization, conversations were frequently confined to broader or admonitory viewpoints, mainly centered on law and societal issues. The legalizing decision seems to have established a favorable setting for investigating more intricate and specialized subjects, such as the therapeutic capabilities of cannabis and its incorporation into wellness regimens.

In addition to reflecting a wider acceptance of cannabis, this move also signifies an expansion of previously restricted discussions. The appearance of frames associated with tourism, celebration, and community effects highlights the favorable stories surrounding the legalization, while also enabling important conversations regarding possible obstacles, such as drug trafficking and public safety.

- *Drug Trafficking.* The frame of “Drug Trafficking” gained more prominence and attention after legalization, with a notable increase in the number of references.
- *Stereotyping.* The frame of “Stereotyping” remained relatively consistent, with no significant change in the number of references.
- *Tourism*. “Tourism” as a frame saw a substantial increase in references after legalization, indicating increased interest in the topic’s impact on tourism.
- *Celebration.* The “Celebration” frame also experienced a significant increase in references following legalization, suggesting a positive perspective.
- *Health and Wellness.* The “Health & Wellness” frame saw a notable increase in references post-legalization, possibly reflecting a growing interest in the health aspects of cannabis legalization.
- *Social and Cultural.* The “Social & Cultural” frame gained more prominence after legalization, with an increase in portrayal.
- *Legal.* The “Legal” frame experienced a substantial increase in YouTube post-legalization clips, indicating a focus on the legal aspects of the topic.
- *Travel Advisory*. This frame also saw a notable increase in clips after legalization, likely reflecting concerns related to travel and safety while carrying cannabis.
- *Competition.* The “Competition” frame experienced a notable increase in post-legalization clips, possibly indicating a more competitive landscape.
- *Human Interest.* The “Human Interest” frame gained significant attention after legalization, with a substantial increase in references.
- *Policy*. The “Policy” frame experienced a remarkable increase in portrayal post-legalization, suggesting a heightened focus on policy-related discussions.

## Discussion

The legalization of cannabis in Thailand has significantly influenced its portrayal as a progressive nation blending tradition with modernity. The findings highlight how positive narratives on YouTube underscore the economic, cultural, and health potential of cannabis legalization, with minimal focus on negative aspects. This aligns with global trends in countries like Canada and Uruguay, where similar reforms have been framed as progressive advancements [26,28].

### Framing narratives on youtube

YouTube serves as a vital platform for reflecting and shaping public opinion on Thailand’s cannabis policy. Dominant frames such as Tourism, Celebration, Health and Wellness, and Human Interest emphasize Thailand’s policy as forward-thinking and beneficial. These frames align with international portrayals of other cannabis-legal nations, suggesting that legalization fosters optimism about economic opportunities, cultural enrichment, and health benefits. Notably, influencers and content creators play a crucial role in constructing these narratives, presenting Thailand as an appealing cannabis tourism destination.

### Comparison with global examples

The analysis reveals parallels between Thailand and other nations like Canada and Colorado, which experienced increased media focus on “marijuana tourism” post-legalization. However, Thailand’s narratives uniquely incorporate emotional and cultural dimensions, highlighting its heritage and modern regulatory strategy. This duality enhances Thailand’s position as a distinct player in the global cannabis discourse.

### Impact of grassroots and official narratives

Thai citizens’ perspectives and grassroots representations significantly contribute to the discourse, complementing the government’s advocacy of cannabis’s therapeutic and economic potential. This is consistent with prior research indicating the Thai government’s proactive efforts in framing cannabis as a national asset [4]. Grassroots involvement contrasts with narratives in other nations, where industry specialists and officials often dominate the discussion [28].

### Absence of negative narratives

Contrary to previous studies suggesting an increase in negative frames like “Drug Trade” or “Stereotype” [42], this study finds no significant rise in such narratives post-legalization. Instead, the deliberate use of positive frames and cautious treatment of potentially controversial aspects have sustained Thailand’s favorable representation.

### Significance of initial reactions

The study underscores the importance of analyzing early public and media responses, which provide critical insights into societal expectations and the initial impact of policy changes. The surge in YouTube content after legalization reflects heightened global interest and optimism regarding Thailand’s decision. These narratives contribute to a broader understanding of how cannabis legalization shapes national and international perceptions.

### Implications for policy and public perception

Thailand’s experience highlights the strategic role of digital media in shaping global narratives. By maintaining positive frames and clear communication of regulatory frameworks, Thailand can further solidify its reputation as a progressive and culturally rich nation. Future policies must leverage these insights to foster sustainable tourism, public health initiatives, and economic growth.

## Conclusion

This study sought to analyze the depiction of cannabis in Thailand after its legalization on YouTube. We conducted an analysis of 57 YouTube video clips using the framework of media framing and nation image. The prevailing storyline was optimistic, highlighting the incorporation of cannabis into Thai agriculture and its therapeutic capabilities. The viability of cannabis as a lucrative commercial commodity also surfaced as a prevailing topic. Nevertheless, a limited number of videos advised visitors about the legal complexities of cannabis consumption, particularly when they came back to their countries of origin. These results indicate an increasing fascination with cannabis tourism in Thailand, as unclear rules seem to have aroused the curiosity of foreign tourists, despite possible legal consequences. Therefore, it is crucial to provide travelers with precise information about cannabis laws to guarantee they are properly educated.

In 2024, almost two years after legalization, the circumstances create an intriguing setting to assess and analyze the regulatory changes that Thailand would encounter in 2022. Given the possibility of a policy reversal towards prohibition, this study becomes all the more pertinent in comprehending how narratives constructed on media platforms could influence public debates and policy choices.

To have a more thorough comprehension, future study could go for bigger sample sizes and broaden the range of data sources, encompassing both conventional and digital media. Analyzing narratives from different countries would also be intriguing, uncovering worldwide perspectives and viewpoints on the legalization of cannabis. It is crucial to examine how shifts in opinion, whether favorable or negative, impact government policies and business and investment choices in the cannabis industry.

In order to avoid any misinterpretations that may damage the country’s global status, we advise politicians and media practitioners to give priority to precise and enlightening depictions of marijuana legalization. Subsequent scholars should further investigate the socio-economic consequences of marijuana legalization in Thailand, providing a more comprehensive interpretation. It is imperative to regularly update this research to accurately represent evolving conditions and tackle possible obstacles, such as the consequences of firm cautions against marijuana goods from different nations and possible governmental reversals.

Although some may argue that our results are outdated, we firmly believe that our research establishes a strong basis for comprehending wider social and policy issues. A retrospective analysis of the narratives that emerged during the initial stage of legalization can offer valuable insights into the dynamics between expectations and realities, as well as how society and governments should address forthcoming circumstances. This study functions as both a historical documentation and a reference for more profound analysis and contemplation of cannabis policy in Thailand and other regions.

## Supporting information

S1 File Supporting File. (DOCX)

## References

[1] Prasad BD. Content analysis: A method in social science research. In: Lal Das DK, Bhaskaran V, editors. Research methods for social work. 2008. p. 173–193. doi: 10.13140/RG.2.1.1748.1448

[2] Neuendorf KA. The content analysis guidebook. Thousand Oaks, CA: Sage Publications; 2002.

[3] Entman RM. Projections of power: Framing news, public opinion, and U.S. foreign policy. Chicago: The University of Chicago Press; 2004

[4] Kalayasiri R, Boonthae S. Trends of cannabis use and related harms before and after legalization for recreational purpose in a developing country in Asia. BMC Public Health. 2023;23(1):911. doi: 10.1186/s12889-023-15883-6 37208663 PMC10197039

[5] Passow T, Fehlmann R, Grahlow H. Country reputation — from measurement to management: the case of Liechtenstein. Corp Reputation Rev. 2005;7(4):309–26. doi: 10.1057/palgrave.crr.1540229

[6] Khan ML. Social media engagement: What motivates user participation and consumption on YouTube? Comput Human Behav. 2017;66:236–47. doi: 10.1016/j.chb.2016.09.024

[7] Yessayan MT. Monarchical nation branding: Queen Rania’s performance of modernity on YouTube. Celebrity Stud. 2015;6(4):430–42. doi: 10.1080/19392397.2015.1087208

[8] Yang S-U, Shin H, Lee J-H, Wrigley B. Country reputation in multidimensions: predictors, effects, and communication channels. J Public Relat Res. 2008;20(4):421–40. doi: 10.1080/10627260802153579

[9] Kunczik M. Transnational public relation by foreign governments. In: Sriramesh K, Verčič D, editors. The global public relations handbook. New Jersey: Lawrence Erlbaum Associates, Inc; 2003. pp. 399–424. ISBN: 9781410607751

[10] Anholt S. From nation branding to competitive identity – The role of brand management as a component of national policy In: Dinnie K, editor. Nation branding: concepts, issues, practice. Oxford, UK: Butterworth-Heinemann; 2008. pp. 22–23. Jin B, Yang H, Kim N. The role of Korean prototypical brand image in shaping country image and quality evaluations: a cross-cultural examination. J Fashion Mark Manag. 2020;25(3). doi: 10.1108/JFMM-10-2019-0232

[11] Fan Y. Branding the nation: What is being branded? J Vacat Market. 2006;12(1):5–14. doi: 10.1177/1356766706056633

[12] Buhmann A, Ingenhoff D. The 4D Model of the country image: an integrative approach from the perspective of communication management. Int Commun Gazette. 2014;77(1):102–24. doi: 10.1177/1748048514556986

[13] Kleber DMS, Juusola K. Open innovation—an explorative study on value co-creation tools for nation branding and building a competitive identity. J Open Innov: Technol Mark Complex. 2021;7(4):206. doi: 10.3390/joitmc7040206

[14] Dinnie K. Nation branding. 2022. doi: 10.4324/9781003100249

[15] Mpofu P. Indigenous media and social media convergence: adaptation of storytelling on twitter, SoundCloud and YouTube in Zimbabwe. J Asian Afr Stud. 2021;57(6):1199–213. doi: 10.1177/00219096211049176

[16] Sobel Cohen M, Riffe D, Kim S. Media and money: a 50-year analysis of international news coverage and U.S. foreign aid. The J Int Commun. 2021;27(2):172–91. doi: 10.1080/13216597.2021.1929391

[17] Thilakarathne KRR, Kumara BTGS, Kuhaneswaran B. Analyzing tourists’ perceptions of tourism destinations using YouTube comments. In: 2021 International Conference on Data Analytics for Business and Industry (ICDABI); 2021 Dec; Sakheer, Bahrain. IEEE; 2021. p. 301–305. 10.1109/ICDABI53623.2021.9655833

[18] Anderson M, Jiang J. Teens, social media & technology 2018. Pew Research Center [Internet]. 2018 [cited 2025 Jan 3]. Available from: http://www.pewinternet.org/2018/05/31/teens-social-media-technology-2018

[19] Holloway D, Green L, Livingstone S. Zero to eight: Young children and their internet use. LSE, London: EU Kids Online [Internet]. 2013 [cited 2025 Jan 3]. Available from: https://eprints.lse.ac.uk/52630/.

[20] Tourism Authority of Thailand. Thailand tourism statistics and projections for 2024. Tourism Authority of Thailand. 2024. Available from: https://www.tourismthailand.org/statistics

[21] Siriwat P, Nijman V. Illegal pet trade on social media as an emerging impediment to the conservation of Asian otters species. J Asia-Pac Biodiv. 2018;11(4):469–75. doi: 10.1016/j.japb.2018.09.004

[22] Ritmontree S, Kanato M, Leyatikul P. The health, economic, and social effects of cannabis use in Thailand. F1000Res. 2019;8:614. doi: 10.12688/f1000research.17391.1

[23] Sommano SR, Tangpao T, Pankasemsuk T, Ponpanumas V, Phimolsiripol Y, Rachtanapun P, et al. Growing ganja permission: a real gate-way for Thailand’s promising industrial crop? J Cannabis Res. 2022;4(1):10. doi: 10.1186/s42238-022-00121-4 35249552 PMC8898406

[24] Rehm J, Elton-Marshall T, Sornpaisarn B, Manthey J. Medical marijuana. What can we learn from the experiences in Canada, Germany and Thailand?. Int J Drug Policy. 2019;74:47–51. doi: 10.1016/j.drugpo.2019.09.001 31525639

[25] Sirita U. Construction and implications of cannabis discourse in Thailand’s Cannabis legalization: a comparative study of english-language traditional and new media. SUT J SOC SCI. 2022;16(2):1–24. doi: 10.55766/aumo7803

[26] Gagnon M, Gudiño D, Guta A, Strike C. What can we learn from the english-language media coverage of Cannabis legalization in Canada?. Subst Use Misuse. 2020;55(8):1378–81. doi: 10.1080/10826084.2020.1741639 32204651

[27] Barata PC, Ferreira F, Oliveira C. Non-medical cannabis use: international policies and outcomes overview. An outline for Portugal. Trends Psychiatry Psychother. 2022;44(Suppl 1):e20210239. doi: 10.47626/2237-6089-2021-0239 34898143 PMC9490937

[28] Cormack P, Cosgrave J. “Enjoy your experience”: Symbolic violence and becoming a tasteful state cannabis consumer in Canada. J Consum Culte. 2021;22(3):635–51. doi: 10.1177/1469540521990876

[29] Boonchutima S. Resistance to change: Thailand’s image as a sex tourist destination. Asian Congr Media Comm J. 2009;1:61–72. Available from: https://www.researchgate.net/publication/260146196

[30] Deng S, Slutskiy P, Boonchutima S. The Chinese media narrative of Thailand as a tourist destination after the legalisation of cannabis. Heliyon. 2023;9(4):e15478. doi: 10.1016/j.heliyon.2023.e15478 37128329 PMC10148097

[31] Alzubi A. Towards digital media and conventional media challenge and opportunity: what to expect. Int J Adv Soc Sci Human. 2023;2(3):152–8. doi: 10.56225/ijassh.v2i3.157

[32] Lewis R. Alternative influence: Broadcasting the reactionary right on YouTube. Data & Society [Internet]. 2018 [cited 2025 Jan 3]. Available from: https://datasociety.net/wpcontent/uploads/2018/09/DS_Alternative_Influence.pdf

[33] Rauchfleisch A, Kaiser J. The German far-right on youtube: an analysis of user overlap and user comments. J Broadcast Electronic Media. 2020;64(3):373–96. doi: 10.1080/08838151.2020.1799690

[34] Buscemi F. Build your nation in 5′25″: social constructions of Italy in official and user-generated videos on YouTube. Social Semiotics. 2016;27(2):129–44. doi: 10.1080/10350330.2016.1158520

[35] Burgess J, Green J. YouTube: online video and participatory culture. Malden, MA: Polity Press; 2009

[36] Briciu A, Briciu VA. Participatory culture and tourist experience: promoting destinations through YouTube. In: Kavoura A, Kefallonitis E, Theodoridis P, editors. Strategic innovative marketing and tourism. Cham: Springer; 2020. p. 47. doi: 10.1007/978-3-030-36126-6_47

[37] Entman RM, Usher N. Framing in a fractured democracy: impacts of digital technology on ideology, power and cascading network activation. J Commun. 2018;68:298–308. doi: 10.1093/joc/jqy028

[38] Tankard JW Jr. The empirical approach to the study of media framing. In: Reese SD, Gandy OH, Grant AE, editors. Framing public life. Routledge; 2001. p. 111–121. doi: 10.4324/9781410605689-12

[39] Mazahir I, Yaseen S, Siddiqui M. International comparison of media coverage on the Fukushima crisis: a comparative content analysis of news media coverage in several countries. Media Educ (Mediaobrazovanie). 2019;59(4). doi: 10.13187/me.2019.4.557

[40] Perko T, Turcanu C. The Fukushima accident: reflection in the media and the public opinion in Belgium. Int J Nucl Gov Econ Ecol. 2012;3(4):291–307.

[41] Ingenhoff D, Calamai G, Sevin E. Key influencers in public diplomacy 2.0: a country-based social network analysis. Social Media Soc. 2021;7(1). doi: 10.1177/2056305120981053

[42] Saingam D. Substance abuse policy in Thailand: Current challenges and future strategies. Stanford: Asia Health Policy Program; 2018. Available from: https://fsi-live.s3.us-west-1.amazonaws.com/s3fs-public/ahppwp_45.pdf

