# Correction: From tradition to progressiveness: Analyzing Thailand’s image on youtube amid post-cannabis legalization

**DOI:** 10.1371/journal.pone.0321708

**Published:** 2025-04-01

**Authors:** Ibtesam Mazahir, Smith Boonchutima, Safeena Yaseen

The first author, Ibtesam Mazahir, is incorrectly noted as the corresponding author. The correct corresponding author is Smith Boonchutima. Smith Boonchutima’s email address is: smith.b@chula.ac.th.

The ORCID iD is missing for the second author. Please see the author’s ORCID iD here:

- Author Smith Boonchutima’s ORCID iD is: 0000-0001-7412-4506 (https://orcid.org/0000-0001-7412-4506).

## References

[1] Mazahir I, Boonchutima S, Yaseen S. From tradition to progressiveness: Analyzing Thailand’s image on youtube amid post-cannabis legalization. PLoS One. 2025;20(2):e0317506. doi: 10.1371/journal.pone.0317506 39919156 PMC11805353

