# Supplementary material for: From tradition to progressiveness: Analyzing Thailand’s image on youtube amid post-cannabis legalization
Source: PLoS One. 2025 Feb 7;20(2):e0317506. doi: 10.1371/journal.pone.0317506 (PMC11805353; doi:10.1371/journal.pone.0317506)
Supplement: S1 File — (DOCX) [file pone.0317506.s001.docx]

# Coding Manual

## 1. Introduction:

### Purpose of the Coding Manual:

This coding manual has been meticulously designed to guide researchers in analyzing YouTube videos that discuss or portray Thailand's international image, especially in the context of cannabis legalization. The objective is to systematically categorize and evaluate the content of these videos to gain a comprehensive understanding of the prevailing narratives, sentiments, and frames.

### Importance of Consistent Coding:

Consistency in coding is paramount to ensure the reliability and validity of the research findings. When multiple coders are involved, consistent coding ensures that each coder interprets and categorizes the content in a similar manner, minimizing subjectivity and bias. This consistency not only enhances the credibility of the research but also ensures that the findings are replicable and can be confidently used for further studies or policy recommendations.

By adhering to the guidelines and categories outlined in this manual, coders will be equipped to systematically evaluate the content, ensuring that the data collected is both accurate and consistent across all videos.

## 2. A. Formal Categories

This part will be filled by the main researcher. The coder will need to double check if the information in the coding sheet is accurate before starting play the video to code the section B. Content Categories.

*Clip Details.* The foundational step in coding involves capturing the basic details of each video clip. This ensures that each video is uniquely identifiable and provides context to the content being analyzed.

*Clip Number.* Assign a unique sequential number to each video clip. This will serve as the primary identifier for the clip throughout the coding process.

*Date Uploaded*. Record the date on which the video was uploaded to YouTube. This helps in understanding the temporal context of the content. Format: DD-MM-YYYY.

*Country of Origin.* Identify the country from which the media or channel originates. This provides insights into potential regional biases or perspectives.

*Name of the YouTube Channel*. Document the exact name of the YouTube channel that uploaded the video. This can offer insights into the credibility, bias, or perspective of the content provider.

*Number of Subscribers.* Note the total subscriber count of the channel at the time of coding. This metric can be indicative of the channel's popularity and reach.

*Genre of the Video.* Categorize the video into one of the predefined genres, such as News/Documentary, Vlog, or Others. This helps in understanding the primary intent or style of the content.

*Video Metrics.* Record the number of views, comments, and likes for the video. These metrics provide a measure of the video's reach, engagement, and audience reception.

*Video Duration.* Document the total duration of the video in minutes and seconds. This can be useful for analyses that consider the depth or breadth of content in relation to video length.

## 2. B. Content Categories:

The content categories delve deeper into the thematic and qualitative aspects of the video clips. This section is crucial for understanding the narratives, sentiments, and perspectives presented in the videos.

*2.1 Topics.*

Each video may touch upon various topics or dimensions. Coders should identify and mark the presence of these dimensions in the video content.

- *Emotional Dimension.* Does the video evoke or discuss emotional aspects related to Thailand's image, especially in the context of cannabis legalization?
- *Physical Dimension.* Are there mentions or discussions about tangible or physical elements, such as landscapes, infrastructure, or cannabis farms?
- *Financial Dimension*. Does the video touch upon economic implications, benefits, or concerns related to cannabis legalization in Thailand?
- *Leadership Dimension.* Are there references to Thai leadership, their decisions, or their stance on cannabis legalization?
- *Cultural Dimension.* Does the video discuss or showcase Thailand's cultural aspects, especially in relation to cannabis?
- *Social Dimension.* Are societal norms, public opinions, or community perspectives on cannabis legalization discussed?
- *Political Dimension.* Does the video delve into the political implications, debates, or decisions regarding cannabis in Thailand?

### 2.2 Tone of the Topics:

For each identified topic or dimension in the video, coders should assess and categorize the tone.

Options:

- *(p) Positive:* The topic is portrayed in a favorable or supportive light.
- *(n) Negative:* The topic is presented with criticisms, concerns, or negative implications.
- *(a) Ambivalent:* The video contains both positive and negative sentiments about the topic.
- *(n) Neutral:* The topic is discussed without any discernible positive or negative bias.
- *(n/f) Not Found.* The topic is not mentioned or discussed in the video.

### 2.3 Tone of the Video Clips:

Beyond individual topics, coders should gauge the overall sentiment or tone of the entire video clip.

- *Examples and Keywords: Coders should refer to specific examples and keywords provided in the manual to assist in determining the video's tone.*

### 2.4 Source of Information:

Identify and document the primary sources or actors quoted or mentioned in the video. Coders should focus on the three most prominently featured sources.

Quoted Actors:

Understanding who speaks in a video provides valuable context and insight into the perspectives and biases that might be present. This section categorizes the potential actors or sources that might be quoted or mentioned in the video clips. Coders should identify and categorize these actors based on the following classifications:

Thai Source:

1. Government Official: This category includes kings, princes, princesses, presidents, ministers, and officials from various government institutions and agencies.
2. Politicians: Members of political parties who are not currently part of the government.
3. NGO: Non-profit, voluntary citizens' groups organized at a local or national level, focusing on specific tasks or concerns.
4. Thai Tourists/Citizens: Ordinary Thai nationals or tourists sharing their perspectives or experiences.
5. Media Source: Journalists or media entities originating from Thailand.
6. Artists: This broad category includes actors, poets, musicians, and other creative professionals.
7. Cannabis Growers, Handlers, and Sellers: Individuals or entities directly involved in the cannabis industry.
8. Security Agencies: This includes the police, defense forces, and other security-related entities.
9. Academicians and Researchers: Experts, scholars, or researchers discussing the topic.
10. Others: Any other Thai sources not explicitly mentioned in the above categories.

International Source:

1. Government Official: This includes presidents, ministers, and officials from government institutions and agencies from countries outside of Thailand.
2. International Politician: Members of political parties from international contexts who are not part of their respective governments.
3. NGO: International non-profit, voluntary citizens' groups or organizations.
4. International Tourists/Citizen: Tourists or citizens from countries other than Thailand.
5. Media Source: Journalists or media entities from countries other than Thailand.
6. Artists: International artists, which include actors, poets, musicians, etc.
7. Cannabis Growers, Sellers, and Handlers: Individuals or entities from the international community involved in the cannabis industry.
8. Security Agencies: International security entities, including police and defense forces.
9. Academicians and Researcher: International experts, scholars, or researchers discussing the topic.
10. Others: Any other international sources not explicitly mentioned in the above categories.

Coders should refer to this appendix when identifying quoted actors in the video clips, ensuring that each actor is categorized accurately based on their role and origin.

### 2.5 Tone of Quotes:

For the statements or quotes from the three primary sources identified, coders should assess and categorize the tone, similar to the tone assessment for topics.

## 3. Frames

Frames are essential narrative structures that shape how information is presented and understood. By identifying the frames used in the video clips, coders can gain insights into the predominant narratives and perspectives surrounding Thailand's image in the context of cannabis legalization.

- Drug Trafficking: This frame delves into the darker side of drug-related activities, highlighting issues like meth distribution, the consequences of drug trafficking, and controversial methods used in the past. Coders should look for discussions or portrayals that emphasize the challenges and dangers associated with drug trafficking in Thailand.
- Stereotyping: Videos using this frame might depict Thailand in a narrow or biased manner, often focusing on sex tourism or presenting tourists in a negative light. Coders should be alert to content that leans heavily on stereotypes, whether about tourists or Thai nationals.
- Tourism: A frame that promotes Thailand as a prime tourist destination, showcasing its attractions, experiences, and unique offerings. Coders should identify content that emphasizes the allure and beauty of Thailand, especially in a post-cannabis legalization context.
- Celebration: This frame accentuates the celebratory aspects of tourism in Thailand, focusing on the entertainment, nightlife, and cultural events. It might also highlight positive reactions to cannabis legalization.
- Health and Wellness: Videos with this frame will discuss the health implications, both positive and negative, of cannabis. This could range from its medicinal benefits to potential health risks.
- Social and Cultural: This frame delves into the societal and cultural perspectives of the Thai people. Coders should look for content that discusses societal norms, cultural shifts, or public opinions related to cannabis.
- Legal: A frame that emphasizes the legal dimensions of cannabis legalization, discussing rules, regulations, and the broader legal landscape in Thailand.
- Travel Advisory: Practical advice for tourists, focusing on safety, legal compliance, and general tips for a pleasant stay in Thailand.
- Competition: This frame compares Thailand's approach to cannabis with that of other countries, highlighting similarities and differences.
- Human Interest: Videos using this frame will focus on personal stories, public reactions, and the broader societal impact of cannabis legalization.
- Policy: This frame discusses the government's stance, policies, and objectives related to cannabis legalization.

For each frame, coders should determine its presence or absence in the video content. The provided examples and descriptions will guide coders in identifying and categorizing these frames accurately.

## 4. Instructions for Coders:

Welcome to the coding process for the study on "Perception of Thailand’s International Image on YouTube." As a coder, your role is crucial in ensuring the accuracy and reliability of the data collected. The following instructions are designed to guide you through the coding process, ensuring consistency and clarity.

### General Guidelines:

- Consistency: Ensure that you apply the coding rules consistently across all video clips. If you're unsure about a particular segment, refer back to this manual or consult with the research team.
- Objectivity: Approach each video without personal bias. Your goal is to code based on the content of the video, not personal opinions or beliefs.
- Attention to Detail: Some videos may contain subtle cues or messages. Pay close attention to both the overt and covert messages in each clip.

### Coding Process:

- Clip Details: Begin by noting down the basic details of the clip, such as the clip number, date uploaded, country of origin, and other metrics. This will provide a context for the rest of the coding process.
- Content Categories: Dive deeper into the content of the video. Identify the topics and dimensions discussed, and determine the tone of each dimension. Use the provided options to classify the tone.
- Tone of the Video Clips: Assess the overall tone of the video. Use the examples and keywords provided in the manual to guide your decision.
- Source of Information: Identify up to three main actors or sources quoted or mentioned in the video. Refer to the Quoted Actors’ List for a list of potential actors and their classifications.
- Tone of Quotes: For each of the main actors identified, determine the tone of their statements or quotes.
- Frames: Identify the frames used in the video. This section requires you to understand the broader narrative or perspective presented in the video. Use the descriptions and examples provided to guide your coding.

### Handling Ambiguities:

If you come across content that is ambiguous or unclear, review the segment multiple times. If still in doubt, consult with a fellow coder or the research team. In cases where content might fit into multiple categories, use your best judgment to select the most dominant or prevalent category. If it's a tie, note down both categories.

### Referring to Examples and Keywords:

The manual provides examples and keywords for various categories and tones. Use these as a reference, but also rely on the broader context of the video. Not all content will fit neatly into the provided examples.

### Documenting Your Work:

As you code, ensure that you document your decisions clearly. If you make any notes or observations outside of the provided categories, ensure they are clearly marked and explained.

Remember, the goal of this coding process is to capture the essence and nuances of each video clip in a structured manner. Your attention to detail and commitment to consistency will greatly contribute to the success of this study.

**Coding Sheet**

**Instructions: Always refer to the coding manual in case of ambiguity or confusion.**

**Clip Title:**

**A: Formal Categories**

| **Variable** | **Category** |  | **Variable** | **Category** |
| --- | --- | --- | --- | --- |
| Clip Number |  |  | Genre of the Video |  |
| Date Uploaded |  |  | No. of Views |  |
| Country |  |  | No. of Comments |  |
| Name of the YouTube Channel |  |  | No. of Likes |  |
| No. of Subscribers |  |  | Duration of the Video |  |

**B: Content Categories B1 & B1a. Topics:** Mark if each dimension is present in the video clip. Code 0 = Absence, 1 = Presence. Also, encircle (1-5) for the tone of the mentioned topic. 1=Positive, 2=Negative, 3=Ambivalent, 4=Neutral, 5= Not Found (N/F)

| **Presence** | **Dimension** | **Tone (1 – 5)** |  | **Presence** | **Dimension** | **Tone (1 – 5 )** |
| --- | --- | --- | --- | --- | --- | --- |
| [ ] | Emotional | 1 2 3 4 5 |  | [ ] | Leadership | 1 2 3 4 5 |
| [ ] | Physical | 1 2 3 4 5 |  | [ ] | Cultural | 1 2 3 4 5 |
| [ ] | Leadership | 1 2 3 4 5 |  | [ ] | Social | 1 2 3 4 5 |
| [ ] | Financial | 1 2 3 4 5 |  | [ ] | Political | 1 2 3 4 5 |

**B2. Overall Tone of the Video Clip**: [ ] 1=Positive, [ ] 2=Negative, [ ] 3=Ambivalent, [ ] 4=Neutral

**B3a. Source of Information**: Identify actors quoted in the video clips. Code the three most mentioned actors. Refer to the Quoted Actors’ List. Code 0 if the actor is not present

| **Source of Information** | **Code (from Quoted Actors’ List)** |
| --- | --- |
| Actor 1 |  |
| Actor 2 |  |
| Actor 3 |  |

**Thai:** 1. Government official – (Name the government personality or party) 2. Politicians 3. NGO 4. Thai Citizens 5. Media Source 6. Artists 7. Cannabis growers, handlers and sellers 8. Law Enforcement Agencies 9. Academicians and researchers 10. Others:
**International:** 11. Government official – (Name the government personality or party) 12. Politicians 13. NGO 14. Thai Citizens 15. Media Source 16. Artists 17. Cannabis growers, handlers and sellers 18. Law Enforcement Agencies 19. Academicians and researchers 20. Others:

**B3b. Tone of Quotes**: Identify the tone of quotes from the actors mentioned.

| **Valence of the Quotes** | **Tone (1-5)** |
| --- | --- |
| quotes_1 | [ ] 1=Positive, [ ] 2=Negative [ ] 3=Ambivalent, [ ] 4=Neutral, [ ] 5=n/f |
| quotes_2 | [ ] 1=Positive, [ ] 2=Negative [ ] 3=Ambivalent, [ ] 4=Neutral, [ ] 5=n/f |
| quotes_3 | [ ] 1=Positive, [ ] 2=Negative [ ] 3=Ambivalent, [ ] 4=Neutral, [ ] 5=n/f |

**B4. Frames**: Identify the frames that appeared in the video clips. Code 0 = Absence, 1 = Presence.

| **Presence** | **Frame** |  | **Presence** | **Frame** |
| --- | --- | --- | --- | --- |
| [ ] | Drug Trafficking |  | [ ] | Legal |
| [ ] | Stereotyping |  | [ ] | Travel Advisory |
| [ ] | Tourism |  | [ ] | Competition |
| [ ] | Celebration |  | [ ] | Human Interest Frames |
| [ ] | Health and Wellness Frame |  | [ ] | Policy |
| [ ] | Social and Cultural Frame |  |  |  |

**List of YouTube Video Samples**

Table . List of YouTube Videos Covering Thailand's Cannabis

| No. | **Title** | **Time** | **Link** |
| --- | --- | --- | --- |
|  | The Hazy Legalization of Marijuana in Thailand | 07:41 | **<https://www.youtube.com/watch?v=x_8iSFoRRfo&ab_channel=TIME>** |
|  | The Stoners Selling Legal Weed in Thailand | 07:29 | **<https://www.youtube.com/watch?v=KAATrEtpai4&ab_channel=VICEAsia>** |
|  | Thailand legalises marijuana but smoking cannabis... | 02:02 | [**https://www.youtube.com/watch?v=EZPWu8NKvjY&ab_channel=TheStraitsTimes**](https://www.youtube.com/watch?v=EZPWu8NKvjY&ab_channel=TheStraitsTimes) |
|  | Thailand becomes the first Asian country... | 02:29 | **<https://www.youtube.com/watch?v=WaYY56RKBLQ&ab_channel=DWNews>** |
|  | Thailand makes pot legal, but smoking discouraged | 01:31 | **<https://www.youtube.com/watch?v=WixjT_UVcRc&ab_channel=AssociatedPress>** |
|  | Thailand legalises cannabis growing and trade... | 01:59 | **<https://www.youtube.com/watch?v=7IFVinoA-7c&ab_channel=BBCNews>** |
|  | Thailand eases cannabis laws but still bans... | 02:16 | **<https://www.youtube.com/watch?v=QL-o-BVaejg&ab_channel=AlJazeeraEnglish>** |
|  | Prisoners In Thailand Released After Marijuana... | 00:54 | **<https://www.youtube.com/watch?v=3Y6YoNX5wVM&ab_channel=NBCNews>** |
|  | EXPLAINED: Thailand Legalises Cannabis... | 05:33 | **<https://www.youtube.com/watch?v=S1NkeL9rSF4&ab_channel=TheProject>** |
|  | All you need to know about the current cannabis situation in Thailand \| This is Thailand | 07:38 | **<https://www.youtube.com/watch?v=JrasIJlEO_s&ab_channel=TheThaiger>** |
|  | How Thailand went from war on drugs to cannabis curries - BBC News | 03:48 | **<https://www.youtube.com/watch?v=znrNrfv__D4&ab_channel=BBCNews>** |
|  | Gravitas: Thailand is giving away 1 million cannabis plants | 04:10 | **<https://www.youtube.com/watch?v=JL_uUM3BIb8&ab_channel=WION>** |
|  | Cannabis in Thailand - Dos and Don'ts \| GMT | 35:34 | **<https://www.youtube.com/watch?v=XkgWHZZtgzs&ab_channel=TheThaiger>** |
|  | Traditional Thai Cannabis Cooking is Back | 06:57 | **<https://www.youtube.com/watch?v=OJkpQsPa73k&ab_channel=Munchies>** |
|  | Thailand starts giveaway of 1 million cannabis plants | 01:58 | **<https://www.youtube.com/watch?v=EiVVRNrSO3E&ab_channel=Reuters>** |
|  | buying weed in Thailand | 17:29 | **<https://www.youtube.com/watch?v=PwOi2ugzFFg&ab_channel=RetiredWorkingForYou>** |
|  | Be Careful About Travel and Cannabis in Thailand | 04:04 | **<https://www.youtube.com/watch?v=2HYVQUkYpaE&ab_channel=IntegrityLegalThailand>** |
|  | Thais React To Cannabis Legalization \| Street Interview | 12:49 | **<https://www.youtube.com/watch?v=wtAQyjt6jhY&ab_channel=AsianBoss>** |
|  | Inside A Thai Cannabis Cafe \| SBS Dateline | 03:05 | **<https://www.youtube.com/watch?v=Cvn8Z15_hoQ&ab_channel=SBSDateline>** |
|  | ‘We don’t welcome those tourists’: Thailand’s health minister discourages weed-smoking visitors | 02:36 | **<https://www.youtube.com/watch?v=VUkbheJvZ9Y&ab_channel=SouthChinaMorningPost>** |
|  | Thailand distributes one million marijuana plants | 00:34 | **<https://www.youtube.com/watch?v=pP_WnS8MiXw&ab_channel=SkyNews>** |
|  | Phuket Rasta Bar Situation, Is THC Legal in Thailand? Phuket Thailand | 34:59 | **<https://www.youtube.com/watch?v=0kBolglZdX0&ab_channel=VloggingADeadHorse>** |
|  | High Fives As Thailand Legalizes Marijuana Possession, Cultivation | 01:11 | **<https://www.youtube.com/watch?v=fC1WIgO95Pk&ab_channel=NBCNews>** |
|  | High season: Bangkok's new cannabis shops could jumpstart tourism this summer | 02:43 | **<https://www.youtube.com/watch?v=RNqcHzjhoEQ&ab_channel=euronews>** |
|  | Thailand Becomes A Weed Wonderland, But With Certain 'Rules' | 00:43 | **<https://www.youtube.com/watch?v=HBxcPpQAx_Y&ab_channel=MIRRORNOW>** |
|  | Weed-smoking Thais celebrate '420' cannabis day AFP | 00:47 | **<https://www.youtube.com/watch?v=DOtuoqppaWc&ab_channel=AFPNewsAgency>** |
|  | Weed-infused bubble tea cashes in on Thailand’s budding cannabis craze | 04:42 | **<https://www.youtube.com/watch?v=PWwyMGcVUms&ab_channel=SouthChinaMorningPost>** |
|  | Government hospitals in Thailand allowed to prescribe cannabis oil for free | 00:59 | **<https://www.youtube.com/watch?v=Nrb9LXYk2Tw&ab_channel=TheThaiger>** |
|  | Celebrations at Thai weed festival after steps to legalisation | 01:01 | **<https://www.youtube.com/watch?v=Zoizpqc522Y&ab_channel=NoCommentTV>** |
|  | NOT Allowed to Smoke CANNABIS in Thailand YET...\| Culture Change. All you NEED to know. | 16:12 | **<https://www.youtube.com/watch?v=UJcOPE9yg5E&ab_channel=ChaiTravel>** |
|  | Bangkok First Cannabis fine dining#cannabis #cannabisthailand | 09:29 | **<https://www.youtube.com/watch?v=FZIMtfjXO-E&ab_channel=SawasdeeThailand>** |
|  | Phuket Thailand Legalize Cannabis/Marijuana/Weed, Phuket Thailand | 11:32 | **<https://www.youtube.com/watch?v=pJkB9T9u5ec&ab_channel=VloggingADeadHorse>** |
|  | BANGKOK THAILAND NIGHTLIFE IN AUGUST 2022 \| LEGAL WEED CANNABIS IN THAILAND TRAVEL \| INDIAN VLOG | 12:37 | **<https://www.youtube.com/watch?v=NIXa4qr9vhk&ab_channel=BucketListVP>** |
|  | Cannabis Scene in Thailand \| The Start of a New Era | 09:17 | **<https://www.youtube.com/watch?v=Pzi8lbbMUKY&ab_channel=TravelsonToast>** |
|  | PRETTY HIGH CANNABIS DISPENSARY (THAILAND) | 02:05 | **<https://www.youtube.com/watch?v=wxRcBW8AkXY&ab_channel=Younggu>** |
|  | Thailand News Today \| Pattaya Walking Street “Ruined” | 08:02 | **<https://www.youtube.com/watch?v=_Gk_uGc1L4g&ab_channel=TheThaiger>** |
|  | Marijuana LEGAL IN PHUKET? Get high and healthy with the best THAILAND CANNABIS! | 03:01 | [**https://www.youtube.com/watch?v=1CqRQUQSG3U&ab_channel=NatalijaKica**](https://www.youtube.com/watch?v=1CqRQUQSG3U&ab_channel=NatalijaKica) |
|  | 24 Hours on Koh Samet Thailand / Cannabis Restaurant | 25:42 | [**https://www.youtube.com/watch?v=NzkYncL5N54&ab_channel=Eatdrinktravel**](https://www.youtube.com/watch?v=NzkYncL5N54&ab_channel=Eatdrinktravel) |
|  | CANNABIS Update Bangkok - MUE BON @- the Cannabis Museum, SIAM SQUARE | 10:33 | [**https://www.youtube.com/watch?v=NcByl_L6g-c&ab_channel=JBWanders**](https://www.youtube.com/watch?v=NcByl_L6g-c&ab_channel=JBWanders) |
|  | Weed Everywhere at KHAOSAN ROAD Now \| Walk in Bangkok Nightlife 4K🇹🇭 | 12:27 | **<https://www.youtube.com/watch?v=-NQS3YFUIY4&ab_channel=ExploringWithFikri>** |
|  | TOP 3 Rated CANNABIS Shops in PHUKET Patong \| Thailand Weed | 15:25 | [**https://www.youtube.com/watch?v=ZAujUlCieRQ&ab_channel=Brennybrez**](https://www.youtube.com/watch?v=ZAujUlCieRQ&ab_channel=Brennybrez) |
|  | Ganja is on Sale at Maejo University Chiang Mai where you can buy 5 Cannabis Plants for 150thb | 05:16 | [**https://www.youtube.com/watch?v=R92STdu2O3I&ab_channel=GoodLifeinChiangMaiThailand**](https://www.youtube.com/watch?v=R92STdu2O3I&ab_channel=GoodLifeinChiangMaiThailand) |
|  | 4/20: Origins of marijuana holiday explained | 04:48 | **<https://www.youtube.com/watch?v=qAMsZdnp5Sc&ab_channel=WGNNews>** |
|  | Selling Weed in Thailand: Copper Pipe, Koh Samui #Interview | 11:12 | **<https://www.youtube.com/watch?v=B1epcOsFY50&ab_channel=ManandDog-Samui%28ThairishSun%29>** |
|  | Immigration Visa Extension - Koh Samui is Getting Busier - Is Marijuana Legal in Thailand | 12:44 | **<https://www.youtube.com/watch?v=EfUSZ5zdxVM>** |
|  | WELCOME to AO NANG, KRABI, THAILAND \| I SMOKED MY FIRST LEGAL CANNABIS JOINT HERE 🌿🇹🇭🇮🇳 \| PSY GEAR | 26:23 | **<https://www.youtube.com/watch?v=9F7kSuCuVwA>** |
|  | CHINATOWN Bangkok \| Delicious Street Food \| Yaowarat Road \| Khlong Ong Ang \| Weed For Sale | 10:30 | **<https://www.youtube.com/watch?v=yxVA3j2h8RQ>** |
|  | Thailand to ban ALL green cigarettes \| GMT | 44:35 | **<https://www.youtube.com/watch?v=ziQhw2P7CN4>** |
|  | 4K Pratunam Night Market Bangkok Walking Street Tour - Thailand 2022 | 19:13 | **<https://www.youtube.com/watch?v=sga1pvho1V0>** |
|  | CANNABIS CAFE SURIN BEACH MARCH 2022 BEST BEACHES IN PHUKET THAILAND TODAY | 13:42 | **<https://www.youtube.com/watch?v=mQE90cfM6OA>** |
|  | Pattaya , This Is Normal? \| Old Weed Man \| Subscriber Meetup \| Soi Boomrang \| Soi 6 \| Daily Life | 19:23 | **<https://www.youtube.com/watch?v=d2AXdA4YVl4>** |
|  | Can Thailand Still Rely On Tourism To Repair Post-COVID Economy? \| Insight \| Southeast Asia | 47:32 | **<https://www.youtube.com/watch?v=u3mrV5Tj0dY&ab_channel=CNAInsider>** |
|  | ONE NIGHT IN PRATUNAM Bangkok \| SUPERNATURAL NIGHTLIFE \| Ghost Stories \| $25 THC Oil | 14:12 | **<https://www.youtube.com/watch?v=fU56PI2Gffk>** |
|  | Modern Day Slaves of Thailand \| Open Secrets | 33:33 | **<https://www.youtube.com/watch?v=qFXOvHizbd8>** |
|  | Trying the Wim Hof Method Workshop in Bangkok, Thailand | 14:33 | **<https://www.youtube.com/watch?v=TzwLqbI4aQ8>** |
|  | Khaosan Road - Bangkok Nightlife | 19:38 | **<https://www.youtube.com/watch?v=1_ievGD4lPM>** |
|  | 🟢4K🟢 🇹🇭 Thailand Bangkok Khaosan Road, Night Street Walking Tour 2022 - 4K 60fps | 58:38 | **<https://www.youtube.com/watch?v=iNC5HY3I1TY>** |
| Sum |  | 11:42:33 |  |
